# Research on the dynamic tensile characteristics and surface crack evolution of coal under impact loading

**DOI:** 10.1038/s41598-024-64342-8

**Published:** 2024-06-10

**Authors:** Ziping Wang, Shengwei Li, Yi Zhang, Peiwen Qi, Youyou Zhang, Yuanjing Chen, Yexue Li, Gang Zeng

**Affiliations:** 1 School of Civil Engineering and Architecture, Hubei University of Arts and Science, Xiangyang, 441053 Hubei China; 2 State Key Laboratory of Hydraulics and Mountain River Engineering, College of Water Resources and Hydropower, Sichuan University, Chengdu, 610065 China

**Keywords:** Coal, Impact load, Dynamic tension, Surface cracks, Fractal dimension, Coal, Civil engineering

## Abstract

The tensile properties of coal under dynamic loading are important mechanical characteristics of coal and are highly important for controlling coal rock stability under impact loading conditions, selecting blasting engineering parameters, and studying the mechanism of rockburst disasters. To investigate the dynamic tensile failure process of coal subjected to impact loading, this study used high-speed photography and digital image correlation technology to capture the dynamic tensile failure of coal under impact loading. The dynamic tensile evolution was quantitatively analyzed from the beginning of coal sample being loaded to failure. The captured images of the coal were processed, and the fractal dimension was used to quantitatively describe the evolution of the coal surface cracks under impact loading. The following conclusions were drawn from the experimental results: (1) An empirical formula was established to describe the dynamic tensile strength characteristics of coal under different loading rates. (2) Under impact loading, the maximum strain of a Brazilian disc coal sample first appeared at the contact end between the sample and the incident rod. (3) Under impact loading, a Brazilian disc coal sample cracked from the center of the sample outward, and the crack subsequently extended toward both ends. The fractal dimension of the crack exhibited a power function relationship with time, and the variation range of the fractal dimension of the crack was 1.05–1.39.

## Introduction

China’s natural energy reserves have the basic characteristics of “poor oil, less gas, and relatively rich coal”^1^. In 2023, coal consumption accounted for 55.3% of China’s total energy consumption. However, it is predicted that this proportion will be approximately stable at 50% by 2050^2^. Under the influence of high stress at deep depths, coal has high elastic potential energy, which increases the possibility of coal and gas outbursts and rock bursts when subjected to external dynamic damage^3–6^. Therefore, investigating the dynamic mechanical characteristics of coal under high-speed impact is very important for predicting and preventing the occurrence of dynamic disasters in coal mines.

Coal has complex physical and mechanical properties, and its tensile strength is far less than its compressive strength^7^. The tensile properties of coal blocks are considered in engineering practice and design. The tensile strength of coal under impact loading is an important parameter for investigating the dynamic tensile properties of coal. The Brazilian splitting test method is typically used to test the dynamic tensile strength of coal. Many studies have conducted relevant research on the dynamic tensile properties of coal subjected to impact loading. Zhao et al.^8,9^ extensively investigated the dynamic tensile strength of coal under different bedding directions and dry and saturated water states using numerical simulations and experiments. They also conducted a preliminary analysis on the dynamic splitting and surface strain field changes of the samples using high-speed cameras and digital speckle image analysis methods. They found that bedding has a significant effect on the dynamic tensile properties of coal. When the bedding was not parallel or perpendicular to the loading direction, the sample exhibited a combination of matrix stretching and bedding shear failure. Compared with the coal samples in the dry state, the coal samples in the saturated state had a stronger rate correlation. Compared with the impact velocity, the bedding direction had a smaller effect on the dynamic tensile strength of coal. Liu et al.^10^ conducted static and dynamic Brazilian splitting tests on coal considering the bedding direction using an MTS815 rock mechanics testing system and a split Hopkinson pressure bar (SHPB). Their results revealed the obvious anisotropy of coal and that the tensile strength of coal in the parallel bedding direction is greater than that in the vertical bedding direction. Therefore, the tensile strength and deformation characteristics of coal exhibit obvious strain rate effects. Han et al.^11^ used SHPB to conduct an experimental study on the evolution of the impact splitting deformation field and crack propagation characteristics of a Brazilian disc coal sample subjected to impact loading. Their results revealed that, in the dynamic tension test of the Brazilian disc, the local crushing zone first appeared at the loading locations at both ends of the coal sample, and the main crack was then generated from the two sides of the region and propagated along the radial loading direction until it reached the other end. Zhu et al.^12^ investigated the dynamic tensile properties of Brazilian disc samples of hard coal in dry and saturated states using SHPB. They found that within the investigated range of impact velocities, the tensile strength of coal in the saturated state was lower than that of dry samples. Hao et al.^13^ conducted dynamic and static loading tests on Brazilian disc coal samples using an improved SHPB and investigated the effects of the static axial prestress and loading rate on the dynamic tensile strength and crack propagation characteristics of coal. They found that the dynamic indirect tensile strength of coal samples first increased and then decreased as the static axial prestress increased. Gong et al.^14^ conducted Brazilian disc splitting tests on easily explosive coal samples under impact conditions using an SHPB and investigated the effects of impact velocity, bedding angle, and saturated water on the total absorbed energy density, total dissipated energy density, and damage variables of the coal samples. Wei et al.^15^ conducted dynamic tension tests on Brazilian disc specimens of coal with different water contents, recorded the dynamic tensile failure process of coal using a high-speed digital camera system, and determined the effect of water content on the dynamic mechanical properties of coal. Ai et al.^16^ produced 24 sets of Brazilian disc coal samples with vertical and horizontal bedding and conducted experiments on them using an SHPB. Their experimental results revealed that the bedding direction has not only a significant effect on dynamic mechanical properties such as the tensile strength, strain rate, and strain energy but also a significant effect on the crack propagation path. Li et al.^17^ used a SHPB to conduct a dynamic Brazilian splitting experiment on coal, discussed the dynamic tensile behavior of coal, and found that the evolution forms and damage modes of coal under different impact velocities are similar. Although many studies have been conducted on dynamic tension tests of coal, the main focus has been on qualitative analysis, and a formula for the variation in dynamic tensile strength with loading rate has not yet been derived.

Extensive research has been conducted on the evolution of coal fractures under static loads using technologies such as high-speed photography^18^, real-time CT scanning^19,20^, and scanning electron microscopy^21^, which are widely used in coal fracture evolution research. Currently, real-time CT scanning, scanning electron microscopy, and other technologies cannot be applied to the study of coal fracture evolution under impact loading owing to the high strain rate of impact loads. The impact failure process of coal under dynamic impact is mainly recorded using high-speed cameras and then analyzed by combining image processing and other technologies suitable for detecting coal fractures. Gao et al.^22^ used high-speed cameras to capture the failure process of coal samples under dynamic compression, and image processing was used to extract the cracks from the sample images captured via high-speed photography. The box fractal dimension was used to quantitatively describe the cracks in the coal samples, and the trend of the variation in the fractal dimension of the dynamic cracks in the coal samples under impact loading with the loading rate was obtained. Hao et al.^13^ conducted dynamic tension experiments on coal and recorded the crack evolution of coal under combined dynamic and static loads using high-speed cameras. Their research revealed that the impact velocity affects the crack propagation mode, while static axial prestressing affects the crack propagation direction. Li et al.^18^ used two high-speed cameras and digital image correlation (DIC) technology synchronized with acoustic emission monitoring to record the fracturing of coal during a dynamic Brazilian disc experiment in real time. Zhang et al.^23^ investigated the mechanical response and crack evolution of coal prone to outburst under dynamic disturbance and the influence of a single prefabricated crack using an improved SHPB, a super-dynamic strain acquisition system, and a high-speed camera system. Although many studies have conducted relevant research on the dynamic fracture evolution of coal under impact loading, further systematic research must be conducted on the dynamic tensile characteristics, strain evolution during dynamic tensile failure, and surface crack evolution after the failure of a Brazilian disc specimen of coal.

This study used an improved SHPB to conduct dynamic indirect tension experiments on Brazilian disc specimens of coal. High-speed photography and DIC technology was used to record the dynamic tensile failure process of coal under impact loading. An empirical formula was established to describe the dynamic tensile strength characteristics of coal under different loading rates. Quantitative analysis was conducted on the dynamic tensile evolution of coal from the beginning of loading to failure, and the images of the fractured coal captured by the high-speed cameras were processed. The fractal dimension was considered to quantitatively describe the evolution of surface cracks in coal under impact loading. The fractal dimension of surface cracks in Brazilian disc coal samples subjected to impact loading was obtained as a function of time.

## Methods

### Sample preparation and experimental methods

**Figure 1:**
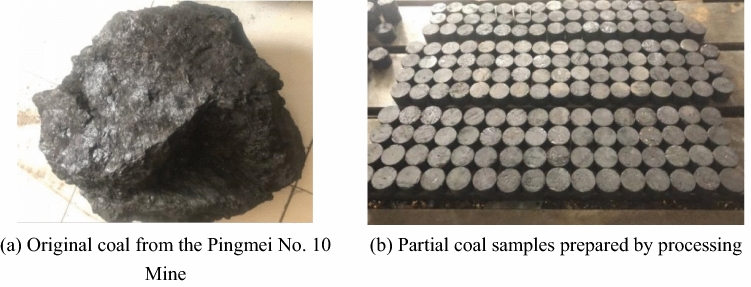
Coal samples.

The selected coal sample was a coal block cut by a coal cutting machine on site in the Pingmei Ji-24130 rock protection layer working face. According to the site conditions and geological reports, the characteristics of the coal samples from this area are the same as those of coal samples from the working face. To facilitate the drilling and preparation of the indoor coal samples, the size of the coal blocks in this study was approximately 25 cm × 25 cm × 20 cm. After the coal samples were collected, they were wrapped in plastic film, boxed, and transported to the laboratory for further sample preparation. To eliminate the influence of bedding on coal properties, the drilling direction is uniformly parallel to the bedding direction for drilling and sample preparation. Figure 1a shows the original coal, and Fig. 1b shows the partially processed coal samples.

To investigate the dynamic failure process of coal under impact loading, this study used high-speed photography combined with DIC technology to record the dynamic failure process of coal under impact loading. Quantitative analysis of the dynamic strain evolution of coal during the entire loading stage from stress to failure was conducted through DIC. The images of the coal captured by high-speed cameras were analyzed, and the fractures were extracted using image processing methods. The fractal dimension was used to quantitatively describe the fracture evolution of coal subjected to dynamic tension and compression under impact loading.

**Figure 2:**
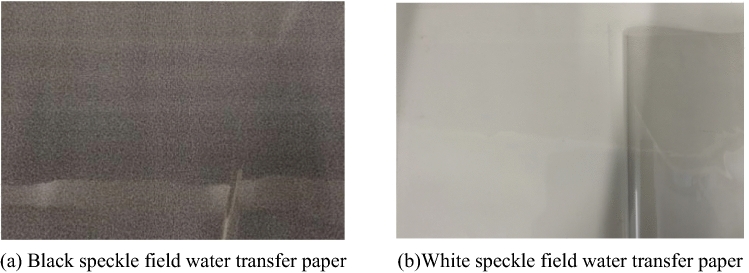
Water transfer paper.

Because DIC tracks the movement and deformation of the measurement area, mainly through the surface speckle characteristics of the object, the quality of the surface speckles of the object is related to the effectiveness of the measurement results. Currently, methods such as heat transfer printing, template engraving, and water transfer printing are often used for the production of digital speckle fields. The digital speckle field of water transfer printing mainly includes three parts: the transparent protective layer of the upper layer, the hydrosol sticker of the lower layer, and the printed digital speckle field between the transparent protective layer and the hydrosol sticker. The thickness of the water transfer paper is not more than 0.5 mm, and the film thickness of the speckle field is only approximately 35 μm, which does not affect the sample’s deformation. When creating the speckle field, water transfer paper is attached to the surface of the object to be tested. After a small amount of water is sprayed on the water transfer paper, the digital speckle field in the middle is close to the surface of the sample, forming a digital speckle field on the surface of the test sample. Previous research has shown that, compared with a speckle field with a natural texture, a water-transferred speckle field has obvious advantages in terms of calculation accuracy and speed. Therefore, in this experiment, the improved water-transferred speckle field method was used to prepare the speckles on the surface of the sample. The black speckle field water transfer paper is shown in Fig. 2a. The white speckle field water transfer paper is shown in Fig. 2b.

**Figure 3:**
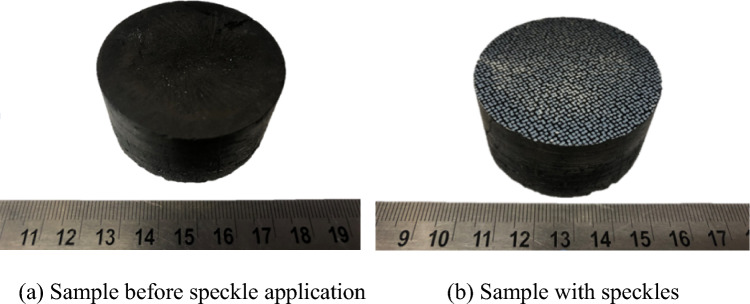
Water transfer printing for speckle creation.

Because the coal sample is black, to obtain a clearer speckle field, for the water transfer paper designed in this experiment, the bottom layer was black, and the digital speckle field in the middle was white. Thus, speckle production on the surface of the coal was completed, as shown in Fig. 3.

### Static mechanical test on coal

**Figure 4:**
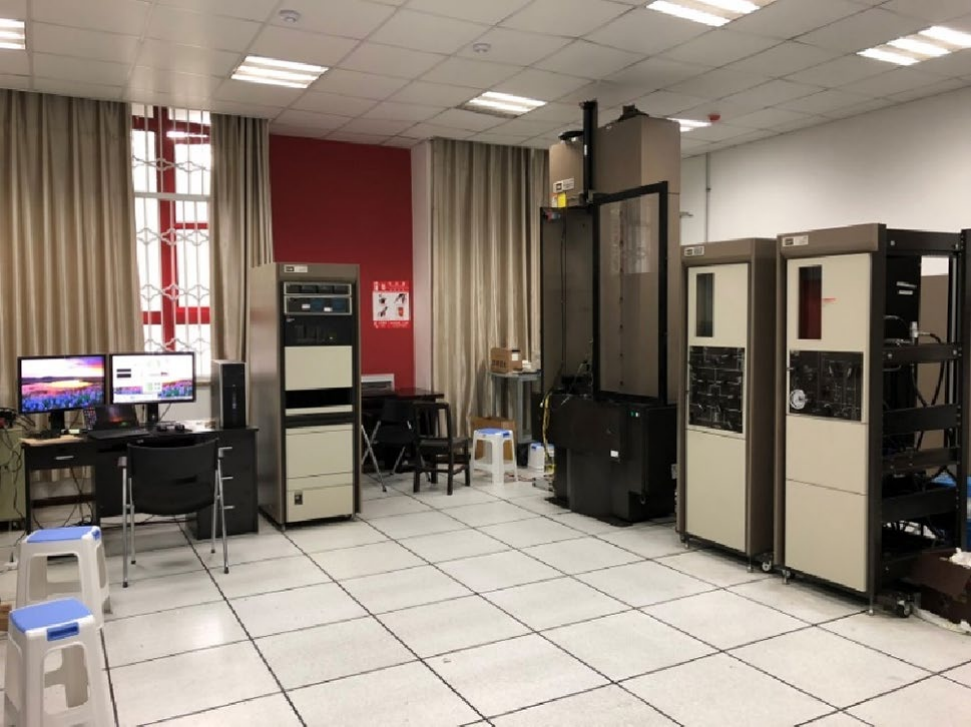
MTS815 Flex Text GT rock mechanics test system.

The static mechanical properties of the coal were determined using the MTS815 rock mechanics test system produced by MTS in the United States and introduced by Sichuan University, as shown in Fig. 4.

The test functions of the MTS815 rock mechanics test system include normal high temperature and high pressure, static, pore water pressure, and acoustic emission positioning^24^. The test options include uni- or triaxial compression, direct or indirect tension, three-point bending, and pure bending. The tests were completed, and the test accuracy and range satisfied the requirements of various test analyses.

### Dynamic mechanical tests on coal

**Figure 5:**
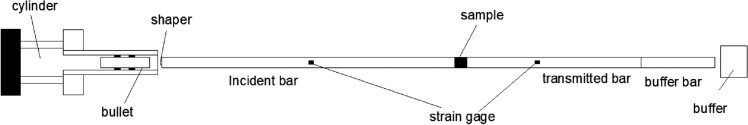
Split Hopkinson pressure bar.

Traditional SHPB mainly include three parts: a bullet, an incident bar and a transmission bar^25^, as shown in Fig. 5. During the experiment, the coal sample was placed between the incident bar and the transmission bar. Driven by high-pressure nitrogen, the bullet hits the incident bar to generate an incident wave, which propagates through the incident bar. When the incident wave reaches the contact surface between the incident bar and the sample, part of it is reflected to form a reflected wave propagating backward, while the other part forms a wave transmitted through the sample and propagating along the transmission bar^26^. The strain signals of the incident wave, reflected wave, and transmitted wave are collected by the strain gauge on the bar, and the stress–strain relationship of the sample can be obtained using one-dimensional stress wave theory.

The forces P_1_ and P_2_ at the ends of the specimen can be calculated using the incident wave strain signal, the reflected wave strain signal, and the transmitted wave strain signal collected by the strain gauge^27^ as follows:

1 $$P_{1} = EA(\varepsilon_{i} + \varepsilon_{r} )$$

2 $$P_{2} = EA\varepsilon_{t}$$

where E is the elastic modulus of the rod and A is the area of the rod.

**Figure 6:**
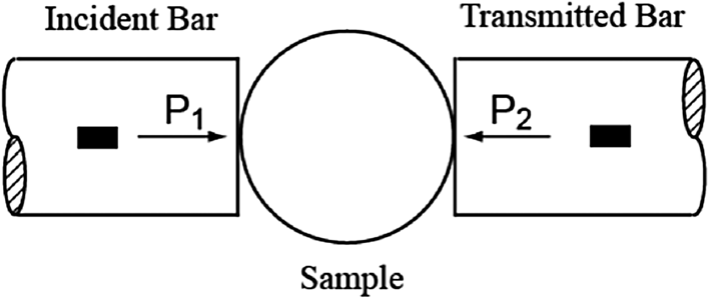
Force on both ends of the specimen in the dynamic tension test.

The dynamic tensile strength of coal during dynamic tensile loading is expressed as follows^28^:

3 $$\sigma_{t} = \frac{2P}{{\pi BD}}$$

where P is the load at both ends of the specimen and B and D are the thickness and diameter of the specimen, respectively. The force on both ends of the specimen in the dynamic tension test is shown in Fig. 6.

**Figure 7:**
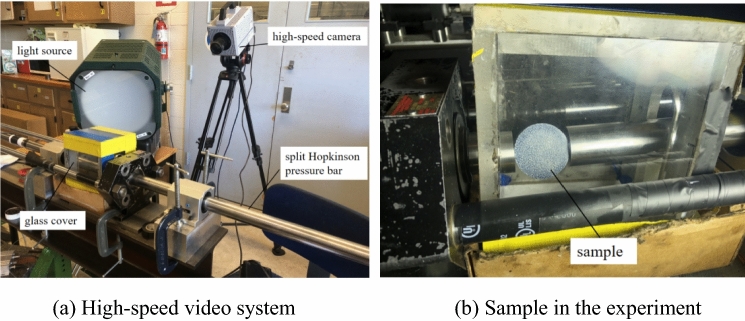
High-speed video system used in the experiment.

In the experiment, a high-speed camera system was used to capture images of the coal under dynamic loading, as shown in Fig. 7^22^. The light source employed high-efficiency film and a television lamp. To facilitate the analysis, high-speed photography and an oscilloscope were used synchronously for recording. The incident strain signal was used to trigger the high-speed camera to start capturing when the incident wave was transmitted to the strain of the incident rod, and the dynamic changes in the coal during the experiment were captured.

In the dynamic compression experiment on coal, when the incident wave was transmitted to the strain gauge of the incident bar, the high-speed camera was triggered to start capturing images, and thus, images of the coal sample under high-speed impact were obtained. The multiscale two-dimensional DIC system developed by the Institute of Mechanics of Southeast University was used to calculate and analyze the measurement results, and the displacement field and strain field of coal under dynamic compression and dynamic tension were obtained at different impact rates. Because the DIC system stops calculating after the sample is destroyed, image processing was performed on the damaged sample to extract the crack, and the crack propagation of coal under dynamic impact was further analyzed.

## Results

### Static tensile strength of coal

**Figure 8:**
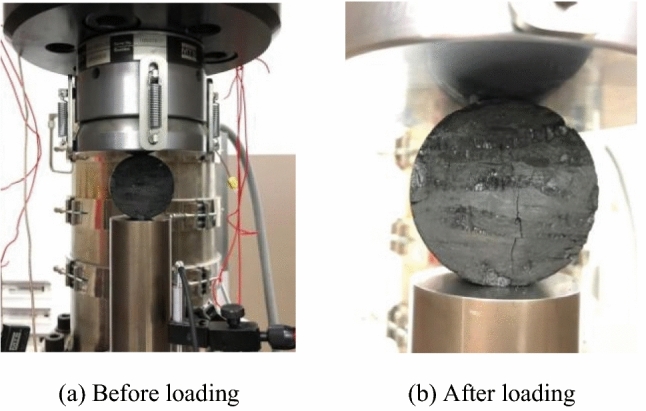
Static Brazilian splitting test on coal.

The shape of the sample after installation and loading failure is shown in Fig. 8. Considering the shape of the coal sample after failure, under external loading, the coal sample started to crack from the center, indicating the effectiveness of the static Brazilian disc test on the coal.

The static tensile strength of the coal was determined to be 0.55 MPa through testing, as presented in Table 1. These results provide a reference for subsequent investigations of the dynamic mechanical properties of coal.

**Table 1:** Static tensile strength of coal.

Numbering	Load force/N	Peak strength/MPa
T-1	1157.86	0.57
T-2	1114.23	0.56
T-3	1152.31	0.53
Mean value	1141.47	0.55

### Dynamic stress equilibrium

**Figure 9:**
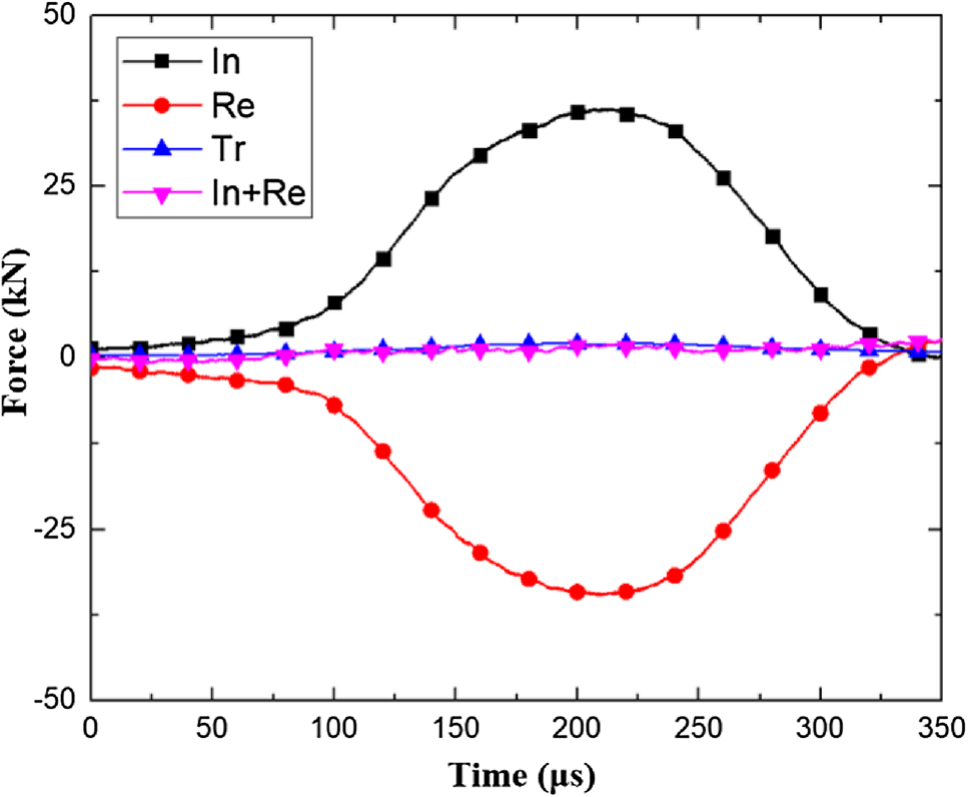
Stress equilibrium diagram.

The stress balance of the sample was tested, and the stress balance diagram under dynamic tensile conditions is shown in Fig. 9. During the dynamic loading process, the dynamic loads at both ends of the coal sample were approximately equal, which satisfied the dynamic stress balance. The method of calculating the loading rate is similar to that of calculating the loading rate in the process of dynamic compression.

### Dynamic tensile strength characteristics of coal

The dynamic tension testing of coal was carried out under different loading rates via Brazilian disc impact tests.

**Figure 10:**
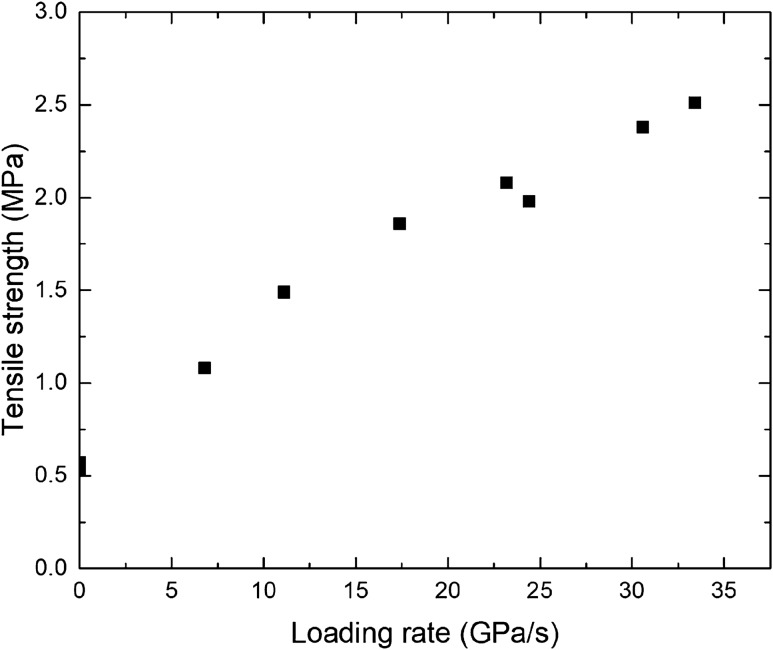
Tensile strength of coal under different loading rates.

Figure 10 shows the tensile strength distribution of coal under different loading rates. The dynamic tensile strength of coal has a significant rate correlation. The loading rate exhibits an obvious nonlinear increase; the rate of increase exhibits a decreasing trend, where the amplitude of increase gradually decreases. When the loading rate increases to a certain extent, the dynamic tensile strength of coal slowly increases and gradually stabilizes.

**Figure 11:**
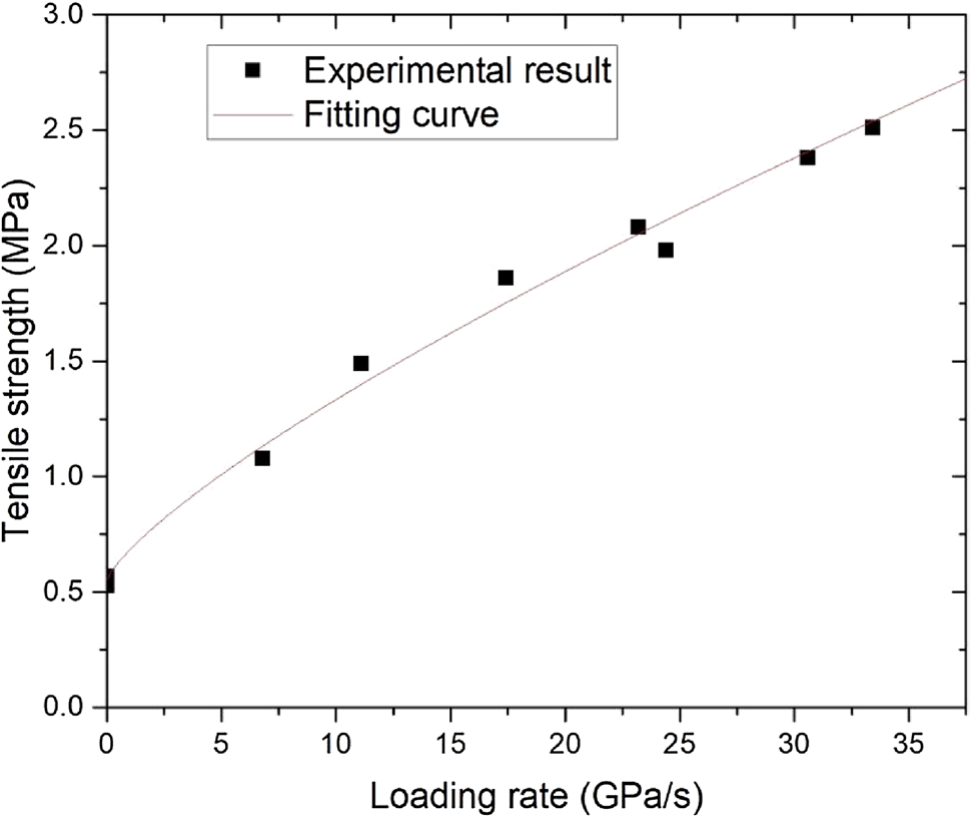
Fitting curve of the dynamic tensile strength of coal under different loading rates compared with the experimental results.

Based on the above experimental results, we can see that the dynamic tensile strength of coal is positively correlated with the loading rate and is distributed in a power function. Then, an empirical formula was established to describe the dynamic tensile strength characteristics of coal under different loading rates.

4 $$\sigma_{td} = \sigma_{ts} + \alpha \left( {\frac{{\dot{\sigma }}}{{\dot{\sigma }_{0} }}} \right)^{n}$$

where the tensile strength of the coal sample, dynamic tensile strength of the coal sample, loading rate = 1.0 GPa/s, reference loading rate, and n are parameters obtained by fitting. In this experiment, the fitting parameters were obtained as follows: α = 0.1323 and n = 0.7723. Figure 11 shows the comparison between the fitting curve of the dynamic strength of coal under different loading rates and the experimental results. The fitting curve is in good agreement with the experimental results, indicating that the empirical formula can reflect the dynamic tensile strength of coal. The empirical formula is of great significance for the stability control of coal and rock under impact loading conditions and the selection of blasting engineering parameters.

### Mode of failure

**Figure 12:**
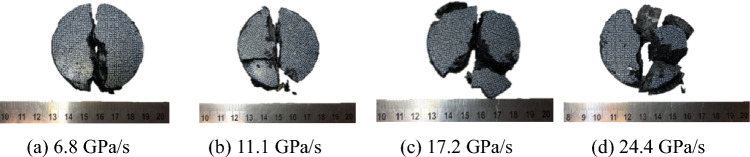
Typical dynamic tensile failure of the specimens.

When the impact load exceeds the tensile strength of coal, fracture will occur. The investigation of the fracture state and final failure mode of coal during the impact process can expand our understanding of the overall fracture, instability mechanism, and development process of coal. In this study, a high-speed camera was used to record the fracture morphology of the coal samples during the impact process. Figure 12 shows the coal sample after failure under different loading rates. The coal sample exhibits significant intermediate splitting failure. Because the high-speed photography and DIC technology were used at a later stage to analyze the strain evolution of coal during the impact process, the surface of the samples was coated with digital speckles. Because the thickness of the digital speckle layer was only approximately 35 μm, its influence on sample deformation during the experiment was ignored. The failure modes shown in the figures accurately reflect the dynamic failure characteristics of coal under different impact rates.

As shown in Fig. 12, when the loading rate was relatively low, the fragments that formed by the fracture of the sample were larger. As the loading rate gradually increased, the degree of fragmentation increased, the number of large fragments gradually decreased, and the number of small particles gradually increased. In other words, as the loading rate increased, the tensile failure of the coal samples became more severe, indicating that the energy absorbed by the coal samples for fracture dissipation increased and that fracture surfaces increasingly formed inside the coal; therefore, the fragmentation became increasingly finer.

## Discussion

### Analysis of the strain evolution in a dynamic tension test

**Figure 13:**
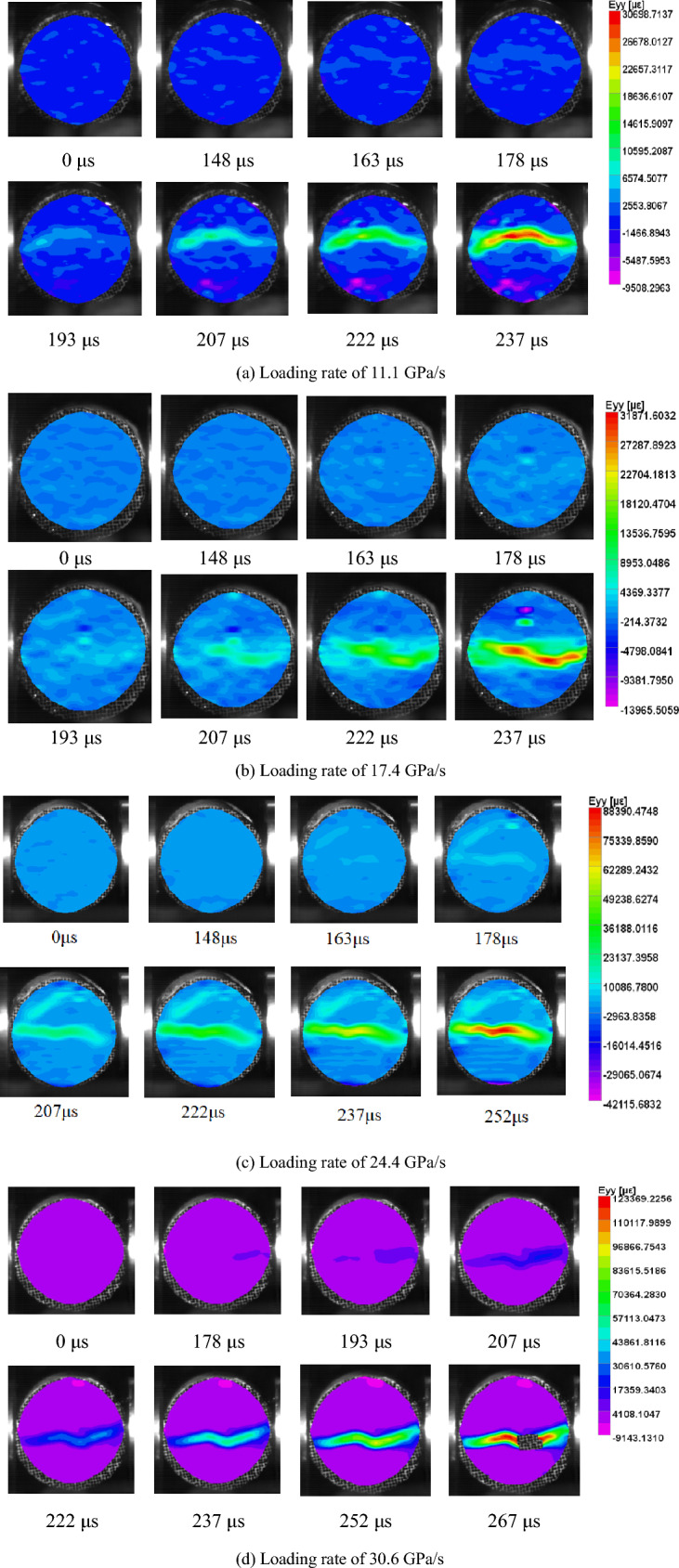
Evolution of the dynamic tensile strain field of coal under different loading rates.

To investigate the dynamic tensile properties of coal under impact loading, dynamic tension testing was carried out on coal samples under different loading rates using the SHPB system. The dynamic deformation of coal was analyzed using the high-speed photography and DIC. Eight high-speed photographs of each sample were selected for DIC analysis, and the strain field in the y-direction of the coal sample under different loading rates during the loading process was obtained, as shown in Fig. 13.

As shown in Fig. 13, the strain field on the surface of the Brazilian disc coal samples was governed by similar rules under different loading rates. At the initial loading stage, the surface of the sample exhibited a tendency to move. The strain on the sample surface was very small as the loading pressure increased. After 163 μs, the strain at the position along the axis of the sample was significantly greater than that on both sides, exhibiting a trend of upward movement for the upper half of the disc and downward movement for the lower half of the disc. In the beginning, the maximum strain appeared at the contact end of the sample and input rod. As the loading progressed, the maximum strain gradually expanded to the sample and output rod end until the overall strain field was symmetrically distributed in the sample. The maximum strain in the y-direction appeared at the center of the sample. Then, the maximum strain at the center of the sample gradually increased and finally exceeded the failure strain at approximately 267 μs. The sample began to crack from the center, which also confirms the rationality of the dynamic tensile strengths of the coal samples measured in the Brazilian disc experiments. Because DIC can only perform calculations on the sample before failure, when the sample is destroyed, the calculation automatically stops; therefore, only the strain evolution before failure can be obtained. By comparing the strain field evolution images on the surface of coal samples under different loading rates, it can be found that the failure time and maximum strain at failure are different. Owing to the frame frequency limitation of the high-speed camera, an image of the sample at the exact failure time cannot be obtained, and only the approximate failure time can be obtained, as a range. As the loading rate increased, the time of crack initiation at the center of the coal gradually decreased, and the strain at failure did not substantially change. Through these quantitative analyses, we can determine the strain at which coal will fail under impact loading and evaluate the stability and safety of coal under impact loading.

### Surface crack propagation of a Brazilian disc specimen under impact loading

**Figure 14:**
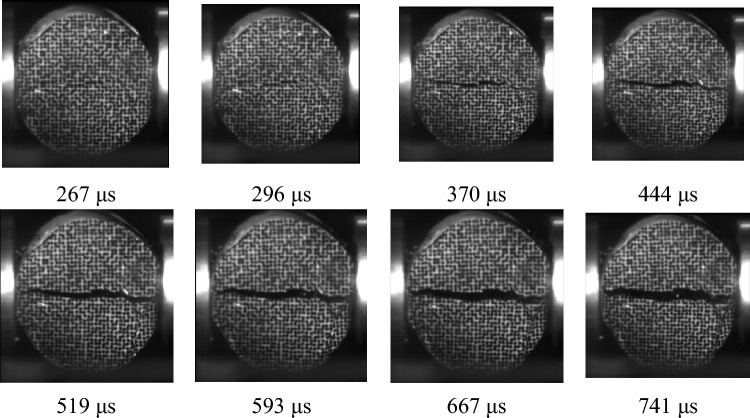
Images captured by high-speed photography during the coal dynamic tension experiment under a loading rate of 24.4 GPa/s.

This study selected a Brazilian disc specimen with a loading rate of 24.4 GPa/s as a typical specimen to analyze the evolution of Brazilian disc cracks under impact loading. An image of coal sample cracking captured by high-speed photography at 741 μs is shown in Fig. 14. The crack starts at 267 s along the center of the sample and gradually expands toward both ends until it penetrates across the entire sample. Then, the upper and lower walls of the sample gradually move toward each other, and the crack width gradually increases. Under dynamic loading conditions, coal samples are subjected to rapid and high-intensity impact forces. This impact force produces complex stress wave propagation and reflection inside the specimen, resulting in inhomogeneity of the stress distribution and dynamic stress concentration. Due to the symmetry of the disc specimen geometry and loading method, the central area becomes a critical area for stress concentration. When the dynamic stress exceeds the tensile strength of coal, failure occurs first at the center of the sample, that is, with the formation of initial cracks. After an initial crack is formed, under the continuous action of dynamic stress, stress concentration and energy release occur at the crack tips. This energy propagates inside the specimen in the form of stress waves, further aggravating the stress concentration at the crack tips, and the crack begins to expand. When the crack extends to the edge of the specimen, it becomes a throughgoing crack.

**Figure 15:**
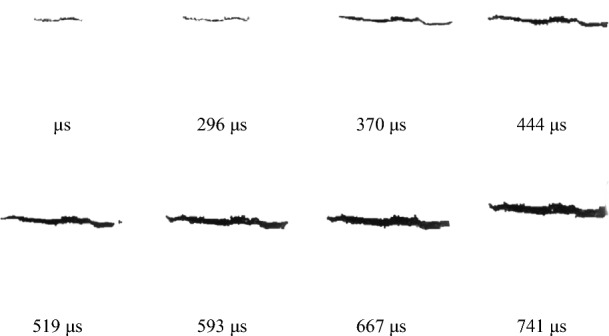
Image of coal fissure evolution.

**Figure 16:**
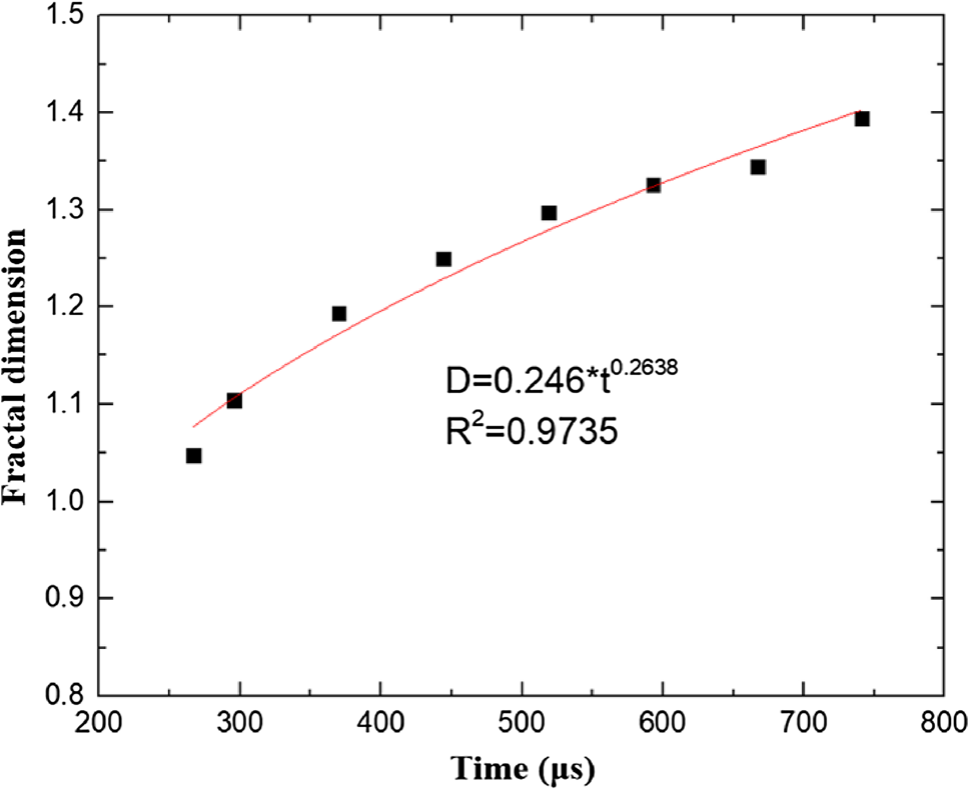
Fractal dimension values of cracks corresponding to different loading times.

By processing the dynamic failure images of coal under different impact velocities, the cracks in the coal were extracted, as shown in Fig. 15. Because the fractal dimension can effectively describe the complexity of the rock mass fracture network, the box dimension method was used to calculate the fractal dimension to quantitatively describe the fractures at different stages after coal failure caused by impact loading. The fractal dimensions at different times during the loading stage are shown in Fig. 16.

As shown in Fig. 16, the fractal dimension of the dynamic tensile cracks in coal changes rapidly in the initial stage of dynamic loading. As time passes, the rate of increase gradually decreases, and the overall increase is exponential, with D = 0.246 × t^0^^.2638^. The range of the variation in the fractal dimension of the cracks is 1.05–1.39. Understanding the crack evolution pattern of coal after dynamic tensile failure will help to more deeply understand the failure pattern of coal under impact loading and help to optimize coal mining processes, such as by the adjustment of blasting parameters.

## Conclusions

This study used high-speed photography combined with digital speckle technology to record the dynamic failure process of coal under impact loading. The dynamic strain evolution of coal during the entire loading stage, from the beginning of loading to the failure of the coal sample, was quantitatively analyzed through DIC, and the cracks were extracted via image processing methods. The fractal dimension was used to quantitatively describe the fracture evolution of coal subjected to dynamic tension and compression under impact loading. The main conclusions drawn from this study are as follows:

(1) There is a significant correlation between the dynamic tensile strength of coal and the loading rate. As the loading rate increased, significant nonlinear growth occurred. The following relationship with the loading rate was identified from the dynamic tensile strength data obtained for the coal:

$$\sigma_{td} = \sigma_{ts} + \alpha \left( {\frac{{\dot{\sigma }}}{{\dot{\sigma }_{0} }}} \right)^{n}$$_._

(2) Under impact loading, the maximum strain of a Brazilian disc coal sample first appeared at the contact end between the sample and the input rod. As the loading progressed, the maximum strain gradually extended toward the sample and the output rod end until the overall strain field was symmetrically distributed in the sample. The maximum strain in the y-direction appeared at the center of the sample, and then the maximum strain at the center of the sample gradually increased until the failure strain was reached and the sample was destroyed.

(3) Under impact loading, a Brazilian disc coal sample cracked from the center of the sample and then extended toward both ends. The fractal number of the crack changed rapidly in the initial stage, and the rate of increase gradually decreased with time. The fractal dimension of the crack exhibited a power function relationship with time, that is, D = 0.246 × t^0.2638^, and the change range of the fractal dimension of the crack ranged from 1.05 to 1.39.

## Data Availability

The authors confirm that the data supporting the findings of this study are available within the article.
